# Acute Diarrhea in Dogs: Current Management and Potential Role of Dietary Polyphenols Supplementation

**DOI:** 10.3390/antiox9080725

**Published:** 2020-08-09

**Authors:** Alessia Candellone, Matteo Cerquetella, Flavia Girolami, Paola Badino, Rosangela Odore

**Affiliations:** 1 Department of Veterinary Sciences, University of Turin, Largo Braccini 2, 10095 Grugliasco, Italy; flavia.girolami@unito.it (F.G.); rosangela.odore@unito.it (R.O.); 2 School of Biosciences and Veterinary Medicine, University of Camerino, Via Circonvallazione 93/95, 62024 Matelica, Italy; matteo.cerquetella@unicam.it

**Keywords:** acute diarrhea, dog, therapeutic management, dietary interventions, polyphenols supplementation, antibacterial resistance

## Abstract

Acute diarrhea is one of the most common reasons why pet owners seek veterinary care for their canine companions. In many cases, signs resolve spontaneously or with symptomatic therapy without a specific cause being discovered. However, life-threatening cases can occur. The etiology is complex, including infectious diseases (endoparasites, virus, bacteria, protozoa, fungal agents) by both zoonotic and non-zoonotic pathogens, dietary indiscretion, endocrine diseases, and stress (e.g., travel or environmental changes). In the last years, the role played by oxidative stress in the pathogenesis of acute and chronic enteropathies, independently from the initial noxa, has been highlighted by many researches in both humans and animals. As a result, a series of dietary antioxidant compounds have been studied for their potential use in the treatment of intestinal inflammation. This review summarizes the traditional therapeutic and nutritional options to manage canine acute diarrhea, highlighting the need to explore the role of oxidative stress and potential antioxidant supplementation, especially polyphenols, during acute diarrheic episodes.

## 1. Introduction

Acute diarrhea (AD) represents one of the most common causes of veterinary consultation in dogs in Western Countries [1]. Diarrhea is a clinical sign characterized by a decrease of fecal consistency, leading to loose or liquid stools, and/or by an increase in the frequency of evacuations in 24 h, with or without fever or vomiting [2]. A classification that takes into account the temporal duration of this phenomenon identifies as “acute” a sign lasting from 3 to 7 days, as “prolonged” the persistence of watery feces from 8 to 13 days, and as “chronic” the appearance of liquid stools for more than 14–21 days [2,3].

Although AD tends to be self-limiting in most cases, and with a mild impact on dog wellbeing [4,5,6], owners commonly ask for veterinary consultation or even resort to self-medication. Etiologic treatments are rarely established because the exact etiology is hardly identified, and only in a few cases (e.g., intestinal parasites) a targeted therapy exists [7]. Hence, a symptomatic medical management with or without dietary change is usually adopted [8,9]. In this context, many cases of uncomplicated AD are treated with antimicrobials, despite the emerging evidence that antimicrobial administration is often unnecessary in dogs with hemorrhagic or uncomplicated AD [10]. In addition, the use of antimicrobials to treat AD is intrinsically linked to the selection and spread of resistant bacteria [11]. A recent retrospective observational study, carried out on a total of 3189 dogs affected by AD, confirmed that antimicrobials are prescribed in 49.7%, gastrointestinal agents (e.g., antacids) in 37.7%, and complementary feeds (e.g., probiotics) in 60.8% of cases [6].

Despite similarities with human disease, where an adequate management to prevent or treat symptoms and the non-use of medications are the bases for the treatment, [2,12,13], standardized protocols and/or official guidelines for proper management of canine AD are quite inconsistent. Remarkably, in 2018 the Federation of International Societies of Pediatric Gastroenterology, Hepatology, and Nutrition (FISPGHAN) Working Group (WG) published Universal Recommendations for the Management of AD in non-malnourished Children, concluding that the routine use of antibiotics is not recommended for the treatment of acute enteropathy [2]. Several in vitro and in vivo studies have investigated the efficacy of therapeutic protocols, alternative to the use of antibiotics [14,15,16,17,18,19], and attention has been focused on the role played by oxidative stress and antioxidant supplementation during acute diarrheic episodes [20,21]. The gastrointestinal (GI) tract is a major site for the generation of pro-oxidants, due to the presence of a plethora of bacteria and food ingredients, that interact with the immune system [22]. In diseases of the GI tract, oxidative stress is due to the imbalance between the microbial-elicited generation of pro-oxidants and the activity of antioxidant defenses [23]. As a consequence, a growing body of evidence suggests that dietary active compounds, such as polyphenols, could exert a protective role against GI tract diseases by modulating inflammatory-related cellular events in the intestine and/or the composition of the microbiota populations [24,25,26,27,28].

Considering the similarities between human and canine AD and according to the paradigm of comparative pathology, it should be emphasized that the disease is characterized by common etiopathogenesis and similar clinical aspects [29]. The growing access to molecular biology techniques for studying the human and canine gut microbiome, and the increasing concern for antibacterial resistance, substantially changed the classification and the therapeutic approach to acute and chronic enteropathies over the last decade [30]. Antimicrobial resistance is a worldwide concern for humans and animals [31]. From this point of view, it should not be underestimated that cohabitation between household pets and humans creates advantageous conditions for interspecific transmission of resistant bacteria [32,33]. Alternative and/or synergistic strategies to traditional drugs in course of acute GI upsets is a desirable approach both in human and veterinary medicine.

This manuscript summarizes current options for the management of canine AD and, by reviewing knowledge on the role played by oxidative stress and antioxidant supplementation in the course of enteropathies, provides the foundation for further research in this field. The attention will be mainly focused on natural dietary compounds, such as polyphenols, due to their well-known potential to directly and indirectly modulate mucosal oxidative stress and gut microbiota.

## 2. Traditional Therapeutic and Nutritional Management

### 2.1. Rehydration Therapy and Electrolytes Replacement

Water is the most important nutrient for dogs with AD, with or without vomiting, because of the potential for life-threatening dehydration due to excessive fluid and electrolyte losses. Oral fluid therapy, containing glucose, amino acids, and electrolytes, is typically reserved for non-vomiting patients with minor fluid deficits, or to supply maintenance fluid requirements in addition to water [34,35]. The physiologic basis for these solutions is the coupled transport of sodium and glucose, and other actively transported small organic molecules. However, such solutions are most beneficial in secretory diarrheas, which are quite uncommon in dogs [36,37]. In a recent study from Tenne et al. [38], the authors tested the hypothesis that a commercially available oral recuperation fluid (ORF), containing prebiotics, omega 6/3 fatty acids, and essential amino acids, may assist in the overall recovery from canine parvovirus enteritis (CPV). Dogs consuming the ORF demonstrated a more rapid return to voluntary appetite and a greater caloric intake, compared with those that consumed water or neither fluid. Moderate to severe dehydration usually needs to be corrected with appropriate parenteral fluid therapy rather than using the oral route. In these cases, crystalloids (i.e., saline, Ringer’s solutions) are frequently administered, and their type and infusion rate shall be selected case-by-case, taking into account the percentage of dehydration, electrolyte/acid-base imbalance and concomitant fluid losses [37]. As regards electrolyte imbalance, hypokalemia is the main predictable consequence of severe acute GI disease because of the elevated concentration of potassium in gastric and intestinal secretions. Mild hypokalemia, hypochloremia, and either hypernatremia or hyponatremia are commonly associated with acute diarrhea and vomiting as well [35]. Electrolyte disorders should be corrected initially with appropriate parenteral fluid and electrolyte therapy, and with the administration of food containing proper levels of minerals (see Section 2.2). As a rule of thumb, at the admission of a patient suffering from AD an IV catheter is usually placed, and fluid resuscitation is performed at the discretion of the clinician. Once fluid deficits are corrected, maintenance IV fluid therapy is administered at a rate of 70–120 mL/kg/day using an isotonic fluid, which may be supplemented with 20 mEq/L of potassium chloride [38,39]. Dextrose supplementation at a continuous rate infusion of 2.5–5% is usually added to the base fluids in hypoglycemic dogs (≤60 mg/dL). Colloidal fluids are administered to dogs with elevated heart rate, unstable blood pressure and/or unresponsiveness to isotonic crystalloids or analgesia. In most critical patients or in those with concurrent cardiac or renal diseases, arterial blood pressure and, ideally, central venous pressure, are constantly monitored during colloids administration, until normalization.

Dogs are usually maintained on IV fluids until they show voluntary food consumption. However, if a patient is enterally fed via an esophageal or nasogastric tube (please see Section 2.2), the amount of water deriving from the liquid diet should be accounted for in the calculation of maintenance needs. Enteral liquid diets for veterinary use contain on average 80% water and have a caloric content of approximately 1 Kcal/mL, or about 0.80 mL of water per Kcal of metabolic energy provided (not counting metabolic water). For example, a hospitalized 10 kg dog fed a commonly available enteral diet, at 100% resting energy requirement, would receive 393 Kcal/day in a total volume of 393 mL, of which 314 mL represents water. Based on the basal water requirement formula from Hensen et al. [39] of 97 × (BW)^0.655 (1.8 mL/kg/h), this patient would require an additional 124 mL/day (approximately 5 mL/h) to be administered parenterally in the form of maintenance fluid. In this situation, 2 mL/kg/h would represent the adequate maintenance fluid rate for appropriate rehydration therapy [39].

### 2.2. Dietary Interventions

Dietary therapy is always part of the treatment plan for canine AD. The goal is to provide a food that meets the dog’s nutritional requirements, allowing for the normalization of intestinal motility and function [35]. Diet for puppies with diarrheal episodes should also meet growth requirements. Several possible nutritional approaches have been traditionally proposed for managing acute small bowel diarrhea in canine patients [35,40]. They may be attempted in any order, provided that the clinical evolution is continuously and carefully monitored. The traditional approach is to first feed a highly digestible (i.e., protein digestibility ≥87%, and fat and carbohydrate digestibility ≥90), low-residue diet with moderate levels of fat (<14% dry matter (DM)basis). This goal can be accomplished by feeding commercial veterinary therapeutic foods formulated for GI diseases or by administering a balanced home-made diet. Small amounts of soluble (i.e., psyllium husks, apple pectin, soy fiber) or mixed fiber sources (pea fiber, beet pulp) may be included in the formulation (≤5% DM basis), as they do not seem to increase fecal volume or impair digestibility [35]. Moreover, especially in the course of acute colitis, dietary soluble fiber content may be increased to normalize intestinal motility, water balance and microbiota. In these cases, a 7% to 15% DM basis amount of fiber, both from soluble and insoluble sources, normalizes transit time and adds indigestible bulk, which buffers toxins, holds excess water and provides intraluminal stimuli to reestablish the coordinated actions of enzyme delivery, digestion and absorption, hormones, neurons, and smooth muscles [35]. As regards protein quality, a high biologic value source should be used (i.e., meat or egg) [35,40]. Due to the possible increase of gut permeability, especially in the course of hemorrhagic AD, some authors empirically suggest the selection of “sacrificial” dietary antigens during recovery, although evidences from controlled dietary trials are lacking [41].

In dogs that have protracted diarrhea, an adjunctive strategy may be the early enteral nutrition, intermittently by the oral route, or continuously by tube feeding. The approach, generally identified as “feeding through vomiting and diarrhea”, has been proposed as an alternative to the traditionally accepted period of bowel rest of 24–48 h, and has been studied prospectively in dogs with parvovirus enteritis [42,43]. The combination of an orally administered highly digestible food (previously incubated with pancreatic enzymes) every eight hours plus total parenteral feeding (TPF) was compared to TPF alone [42]. Dogs in the combined therapy group had a lower mortality rate than those receiving total parenteral nutrition alone, but the intermittent oral administration of food was complicated by nausea and vomiting in 90% of patients. Moreover, the effect of early enteral nutrition using a polymeric enteral food administered continuously by a naso-esophageal tube was evaluated in patients with CPV as compared to dogs held nihil per os (NPO) [43]. Early enteral nutrition resulted in a more rapid clinical improvement, depicted by increased body weight, resolution of vomiting and diarrhea, and a lower mortality rate. The precise mechanisms responsible for these benefits are unknown, but they may include increased protein/caloric intake, more rapid intestinal villous recovery, enhanced integrity of epithelial tight junctions, normalization of intestinal microbiota and enhanced gut immunity [42]. In dogs held NPO for acute gastroenteritis, reintroduction to oral feeding may be accomplished by offering small amounts of a highly digestible canned food formulated for gastro-intestinal disease or monomeric liquid food containing maltodextrins and glutamine. The amino acid glutamine is considered a conditionally essential nutrient for human and animal patients with severe AD, as it is necessary for maintaining gut mucosal integrity [40,44]. Unfortunately, an analytical method for the measurement of glutamine levels in food is not widely available, making the selection of food based on glutamine content unfeasible [45]. Glutamine intake can be increased by orally administering a 2% solution of glutamine in water; 0.5 g of glutamine per kg of body weight should be provided daily. It has also been demonstrated that feeding a monomeric, iso-osmotic liquid food containing maltodextrins (no lactose) plus glutamine to puppies recovering from CPV reduced nausea and vomiting, and subsequently facilitated the transition to feeding other commercial veterinary therapeutic foods [35].

As regards the correction of electrolyte imbalance (hypokalemia, hypochloremia), food specifically formulated for patients with acute gastroenteritis should contain levels of potassium, sodium, and chloride above the minimum allowances for normal dogs and cats. Recommended levels of these nutrients for dogs are 0.8% to 1.1% DM basis of potassium, 0.30% to 0.5% DM basis of sodium, and 0.5% to 1.3% DM basis of chloride [35]. Key nutritional factors for dogs with AD are summarized in Table 1.

### 2.3. Complementary Feeds

Complementary feeds, especially pre- and/or probiotics, are commonly used in managing acute and chronic cases [6,46]. Without colonizing the intestine, bacteria administered with probiotics can modify GI microbiota composition and metabolism—with beneficial effects, such as the counteraction of dysbiosis [47]. In a study performed in dogs with acute uncomplicated idiopathic diarrhea, probiotic administration (*Bifidobacterium animalis* strain AHC7) allowed a shorter resolution time and a lower proportion of patients receiving metronidazole, if compared with a control group (placebo) [48]. Similarly, in dogs with acute self-limiting gastroenteritis, probiotic supplementation (*Lactobacillus farciminis*, *Pediococcus acidilactici*, *Bacillus subtilis*, *Bacillus licheniformis*, and thermo-stabilized *Lactobacillus acidophilus* MA 64/4E) reduced the time for feces normalization with respect to a placebo [49]. In addition, probiotic administration (*Lactobacillus fermentum* VET 9A, *Lactobacillus rhamnosus* VET 16A, and *Lactobacillus plantarum* VET 14A) not only improved stool consistency but was also showed to be associated with a reduction of potentially pathogenic fecal bacteria [50], and (*Lactobacillus plantarum* DSM 24730, *Streptococcus thermophilus* DSM 24731, *Bifidobacterium breve* DSM 24732, *Lactobacillus paracasei* DSM 24733, *Lactobacillus delbrueckii* subsp. *bulgaricus* DSM 24734, *Lactobacillus acidophilus* DSM 24735, *Bifidobacterium longum* 120 DSM 24736, and *Bifidobacterium infantis* DSM 24737) to a decrease of toxigenic *Clostridium perfringens* in dogs with acute hemorrhagic diarrhea syndrome [51]. Interestingly, in a randomized double blinded placebo-controlled clinical trial performed in dogs with AD, probiotics (*Bifidobacterium bifidum* VPBB-6, *Bifidobacterium longum* VPBL-5, *Bifidobacterium animalis* VPBA-4, *Bifidobacterium infantis* VPBI-6, *Lactobacillus acidophilus* VPLA-4, *Lactobacillus plantarum* VPLP-5, *Lactobacillus casei* VPLC-1, *Lactobacillus brevis* VPLB-5, *Lactobacillus reuteri* VPLR-1, *Lactobacillus bulgaricus* VPLB-7) seemed to help in reaching an acceptable fecal consistency more rapidly, than in dogs administered metronidazole or placebo [52]. Despite many studies suggest positive effects following probiotic administration in diarrheic dogs, a recent systematic review highlights the need for much larger randomized controlled studies to support the claim [53]. Finally, the use of adsorbents, such as bentonite-montmorillonite or kaolin, is described in a case of acute enteropathies [35], also being included in complementary feeds.

### 2.4. Antibacterial Drugs

As regards antibacterial administration in canine acute diarrhea, it should be properly evaluated on a case-by-case basis, as it has been recently reported in a chronic disorder [30]. Different molecules (e.g., amoxicillin/clavulanic acid, and metronidazole) have been studied over time in AD, and it is now generally accepted that their use should be mainly reserved for those cases presenting sepsis, or at a clear risk of sepsis (evidence of infection associated to systemic inflammatory response syndrome) [5,6,10,54,55,56]. For instance, it has been shown that the administration of amoxicillin/clavulanic acid in dogs presenting with acute hemorrhagic diarrhea did not significantly improved parameters, such as severity of clinical signs, fecal consistency, and time of hospitalization, if compared with a placebo group [54]. However, it should be reported that metronidazole has been proven to modestly shorten the duration of clinical signs in some dogs with acute non-specific diarrhea, even if it cannot be excluded that similar results could have been achieved through different therapeutic approaches [5]. In addition, according to Unterer and coll. bacteremia is not always significantly present in dogs suffering from idiopathic acute hemorrhagic diarrhea syndrome, in comparison with healthy controls [55].

Judicious use of antibacterials is also needed to limit their impact on GI microbiome. It has been reported that tylosin, commonly prescribed to diarrheic dogs, causes dysbiosis with a reduction in bacterial diversity in healthy dogs [57], similarly to what occurs in dogs with AD [58]. Moreover, changes in the composition of intestinal microbiota can last in some cases for a long time, with modifications persisting up to four years, as it has been reported in human medicine (e.g., clarithromycin plus metronidazole administration) [59].

### 2.5. Miscellaneous

Antacids, antiemetics, gastro-protectants, anti-inflammatory drugs, and analgesics are also variably administered depending on clinical sings, their severity, and their etiology [37]. Finally, although much more evidence is needed, fecal microbiota transplantation (FMT) represents a promising approach to diarrhea. Encouraging results have been obtained in puppies with CPV, in which FMT plus standard therapy was associated with a faster clinical recovery when compared to standard therapy alone [60]. Furthermore, recently FMT was shown to reduce (normalize) the dysbiosis index in dogs presenting acute diarrhea, with significantly better results at two consecutive time-points (7 and 28 days) compared with dogs treated with metronidazole [61].

## 3. Oxidative Stress in Course of AD and Polyphenols Supplementation as a Potential Additional Support Option

### 3.1. Mechanisms of Oxidative Stress-Induced Damage

**Figure 1:**
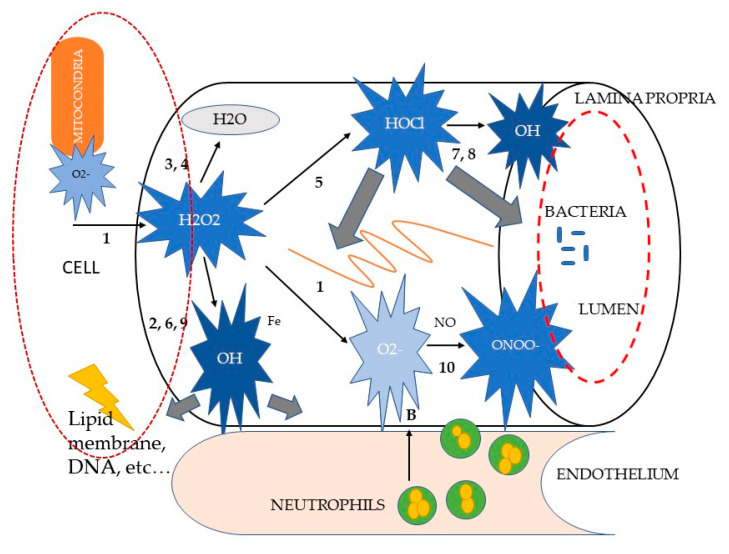
Schematic representation of reactive oxygen metabolite (ROM) reactions in intestinal inflammation, modified from [66]. Letters indicate the sources of ROM (A, neutrophil NADPH oxidase; B, xanthine oxidase; C, mitochondrial NADPH cytochrome p450 reductase). Numbers correspond to the (anti)oxidant reactions cited in the text. The large grey arrows indicate possible ROM targets (membrane lipids, proteins, DNA, matrix blood vessels, bacteria). Fe, ferrous iron; H_2_O_2_, hydrogenperoxide; HOCl, hypochlorous acid; MT, metallothionein; NO, nitric oxide; O_2_^•–^, superoxide anion; •OH, hydroxyl radical; ONOO–, peroxynitrite.

The intestinal mucosal barrier has the primary functions of allowing the passage of nutrients and fluids, as well as of protecting the host from foreign antigens. Its integrity is based on a fine coordination of cell events: proliferation, migration, differentiation, and apoptosis [62]. It has been clearly established, both in humans and in animal models, that during acute and chronic diarrhea the mechanisms responsible of cell turnover are mainly subverted, leading to different degrees of mucosal barrier damage and to clinical manifestations of GI disease [63,64]. The loss of intestinal barrier integrity also promotes abnormal immune and inflammatory responses that are triggered by increased interactions between gut microbiota and the host immune system, a key factor in the development of chronic enteropathies in dogs [27,29,30], such as food-responsive enteropathies (FRE) or those which respond to steroids administration (SRE or immunomodulant-responsive enteropathies IRE) [29,30]. The macrophages lying in the *lamina propria* of the mucosa recognize the luminal microbes that cross the epithelial barrier and multiply within host tissues, acting as antigen presenters for T lymphocytes. Additionally, they enhance the secretion of cytokines and chemokines that attract and activate other immune cells, such as lymphocytes and neutrophils [65]. Thus, the production of selectins and factor VIII by endothelial cells promotes the sequestration of circulating leucocytes at the site of inflammation. Selectins are responsible for the adhesion of lymphocytes and granulocytes to the target tissue, whereas factor VIII promotes the activation of the complement pathway and the kinin cascade, enhancing the intestinal permeability. Macrophages and neutrophils represent the primary source of reactive oxygen and nitrogen metabolites (ROM and RNM, respectively) within the inflamed intestinal mucosa [65,66]. The primary ROM is the superoxide anion (O_2_^•–^)a free radical containing an unpaired electron formed from the single electron reduction of molecular oxygen. It is generated through a variety of sources in both physiological and pathophysiological conditions; however, in the pathogenesis of intestinal inflammation, the major role is played by neutrophils and macrophages, as previously mentioned. These cells, upon interaction with pro-inflammatory agents, undergo a so-called “respiratory burst” [67]. This process involves a sudden stimulus-induced activation of the membrane-bound enzyme nicotinamide adenine dinucleotide phosphate (NADPH) oxidase, which in turn evokes the release of large amounts of ROM. Although it is beyond doubt that O_2_^•–^ generation by phagocytes is essential for an effective host defense against bacterial infection, its continuous overproduction during inflammatory processes may also cause extensive tissue destruction. As much as 1–5% of the total oxygen consumption by normal tissues might be transformed into O_2_^•–^, which makes the mitochondrion the major endogenous intracellular O_2_^•–^ production site. Significant amounts of O_2_^•–^ can also be generated by a variety of endogenous enzyme systems, such as the peroxisomal enzyme xanthine oxidase, which is activated by the reintroduction of oxygen after periods of hypoxia. Despite this enormous production, O_2_^•–^ itself is not considered a particularly reactive intermediate: it does not rapidly cross lipid membrane bilayers and it dismutates spontaneously at physiological pH (see reaction (1), Figure 1).

Paradoxically, the danger of O_2_^•–^ lies in its neutralization. Reaction (1), accelerated by the super-oxide dismutase (SOD) enzyme, is the first of a cascade of other oxidant reactions (see Figure 1), yielding much more powerful ROM, such as hydrogen peroxide (H_2_O_2_), hypochlorous acid (HOCl) and the hydroxyl radical (•OH) [66,67,68,69].

Although H_2_O_2_ has been shown to directly exert non-specific irreversible damage to epithelial cells, it is generally considered as a relatively weak ROM [67]. Its high in vivo reactivity is not only attributed to its stability and diffusibility, but particularly to its ability to react with partially reduced metal ions, such as Fe^2+^ or Cu^+^, to form •OH in the so-called Fenton reaction (see reaction (2), Figure 1). The formation of •OH from H_2_O_2_ can be bypassed through the two-electron reduction of H_2_O_2_2 to water, and catalyzed by catalase (CAT) (reaction (3)) or glutathione peroxidase (GPO) (reaction (4)). Instead of being neutralized to water, H_2_O_2_ can also be metabolized by the enzyme myeloperoxidase to form the potent chlorinating as well as oxidizing agent HOCl (reaction (5)). This reaction is specifically considered to be relevant in inflammatory processes, as the haemoprotein myeloperoxidase is one of the most abundant proteins in phagocytes [66,67,70]. When activated, neutrophils can secrete myeloperoxidase extracellularly. HOCl is estimated to be 100–1000 times more toxic than O_2_^•–^ or H_2_O_2_ and seems to have distinct biochemical targets. For example, it is capable of inactivating essential enzymes, of oxidizing plasma membrane thiol (SH) groups, of disrupting certain protein and plasma membrane functions, and of decreasing the adhesive properties of some extracellular matrix components.

Hydroxyl radical (•OH) is considered the most reactive ROM. In contrast with H_2_O_2_, •OH inactivates the pivotal mitochondrial enzyme pyruvate dehydrogenase, depolymerizes gastrointestinal mucin and directly inflicts DNA damage. •OH is formed from H_2_O_2_ through the Fenton reaction (reaction (2)) or from O_2_^•–^ through another transition metal-dependent reaction, called the iron-catalyzed Haber–Weiss reaction (reaction (6)), [71]. In the last decade, •OH has been shown to be produced via certain alternative, but inflammation-relevant, pathways. They include the generation of •OH during the inactivation of Cu⁄Zn-SOD by H_2_O_2_ and through the interactions between O_2_^•–^ and HOCl (reaction (7)) and reduced iron ions (reaction (8)) and H2O2 and nitric oxide (NO) (reaction (9)), [66]. Again, no known enzyme exists to facilitate the detoxification of •OH. However, •OH-induced tissue damage may be prevented by the binding (“sequestration”) of transition metal ions by, for instance, albumin, caeruloplasmin, ferritin, transferrin, metallothionein, or exogenous antioxidant compounds, such as the polyphenol epigallocatechin gallate, which is the most effective catechin against oxidative stress via hydrogen peroxide and radical scavenging activity [66,67,72].

### 3.2. Role of Polyphenols Supplementation in Course of Intestinal Inflammation

Oxidative stress is one of the major fundamental tissue-destructive mechanisms, through an excessive release of reactive oxygen metabolites (ROM), as described in Section 3.1. Reactive oxygen metabolites can directly cause reversible and irreversible damage to any oxidizable biomolecule. Consequently, they have been implicated in cell or tissue damage of practically every disease, including acute and chronic enteropathies (66). For instance, elevated levels of ROM have been detected in humans affected by inflammatory bowel disease (IBD) and ulcerative colitis (UC), as well as in murine models with acute and chronic colitis [27,66]. Interestingly, oxidative markers have also been investigated in veterinary medicine by analyzing fecal samples, both in healthy hunting dogs during exercise and in dogs with IBD, suggesting different degrees of oxidative stress [67,68]. Consequently, it is reasonable to assume that mucosal damage caused by high levels of ROM may also play a key role in the pathogenesis of acute and chronic enteropathies in dogs.

Given the above, in vivo and in vitro studies [73,74,75,76,77,78,79,80,81] have been performed to elucidate possible protective mechanisms of dietary antioxidant compounds in the treatment of intestinal inflammation and redox imbalance, with particular attention to natural polyphenols. Polyphenols represent a great variety of compounds occurring in fruits, vegetables, and plant-derived products. The research interest in these substances is continuously increasing due to their potential health benefits. Suggested beneficial effects are anticarcinogenic [82,83], antiatherogenic [84,85], antiulcer [86], antithrombotic [87,88], anti-inflammatory [89,90], immune modulating [91], antimicrobial [92,93], and analgesic activities [94]. Traditional antioxidant compounds, such as ascorbic acid and tocopherol, which share with polyphenols some of the aforementioned properties, have been poorly investigated in human and small animal enteropathies. This could be explained by the peculiar mechanism of action and biotransformation processes that distinguish phenolic molecules from other antioxidants. Polyphenols act mainly through their low-molecular-weight metabolites that are potentially more bioactive than parent compounds. Total polyphenol absorption by the small intestine is relatively low (5–10%) in comparison to other macro- or micronutrients; thus, 90–95% of polyphenols transit to the large intestinal lumen and accumulate in a millimolar range. In the colonic lumen, together with conjugates excreted through the bile, they are exposed to the enzymatic activities of the gut microbiota that generate metabolites with a potential greater biological activity than the parent compounds, and constituting the so-called “food metabolome” [25]. The reciprocal relationship between polyphenols and gut microbiota may contribute to host health benefits. Such interaction entails microbial degradation of polyphenols and modulation of gut microbiota by polyphenol metabolites, which in turn inhibit pathogenic bacteria and stimulate beneficial microbes [27,79]. Commensals residing in the gut may improve health by protecting against GI disorders and pathogens, processing nutrients, reducing serum cholesterol, strengthening intestinal epithelial tight cell junctions, increasing mucus secretion, and modulating intestinal immune response through cytokine stimulus [25]. Several phenolic compounds have been recognized as potential antimicrobial agents with bacteriostatic or bactericidal actions. Similarly, they also inhibit bacterial infections of intestinal and urinary tract epithelia [95,96]. Phenolic compounds, such as quercetin, rutin, genistein, (+)-catechin and (−)epicatechin, O-methylgallic acid, gallic acid, and caffeic acid, alter the gut microbiota and, consequently, modify the *Bacteroides/Firmicutes* balance [96,97]. For example, an in vitro study, using a batch-culture fermentation system reproducing the distal region of the human large intestine, suggested that flavan-3-ol monomers, including(−)epicatechin and (+)catechin, may shape the large intestinal bacterial population even in the presence of other nutrients, such as carbohydrates and proteins. Indeed, (+)catechin significantly increased the growth of *E. coli* and members of the *Clostridium coccoides-Eubacterium rectale* group, while it inhibited the growth of *Clostridium histolyticum*. *Bifidobacterium* and *Lactobacillus spp*. remained relatively unaffected [98]. Similarly, the dietary administration of proanthocyanidin-rich extracts in rats for 16 weeks appears able to modify the colonic flora, with a shift from a predominance of *Bacteroides, Clostridium* and *Propionibacterium spp*. to a predominance of *Bacteroides, Lactobacillus* and *Bifidobacterium spp*. [99]. Effects of polyphenols on gut microbiota composition and diversity are summarized in Table 2.

Among polyphenols, quercetin, quercitrin, resveratrol, and rutin represent the most studied compounds in in vivo trials. For instance, quercitrin was tested for acute anti-inflammatory activity in trinitrobenzene sulfonic acid-induced rat colitis from Sanchez de Medina et al. [100]. The inflammatory status was evaluated by myeloperoxidase, alkaline phosphatase and total glutathione levels, leukotriene B4 synthesis, in vivo colonic fluid absorption, macroscopical damage, and occurrence of diarrhea and adhesions. Treatment with 1 or 5 mg/kg of quercitrin by the oral route reduced myeloperoxidase and alkaline phosphatase levels, counteracted glutathione depletion, preserved normal fluid absorption and ameliorated colonic damage at two days. Increasing or lowering the dose of the flavonoid resulted in a marked loss of effect. The acute anti-inflammatory effect of quercitrin seemed to be unrelated to the impairment of neutrophil function and it may be caused by mucosal protection or enhancement of mucosal repair secondary to the increased defense against oxidative insult and/or preservation of normal colonic absorptive activity. The same research group also demonstrated that the beneficial effects of quercitin on trinitrobenzene sulfonic acid colitis arises from an early downregulation of the inflammatory cascade that is associated with amelioration of the disturbances in hydro-electrolytic transport [101]. Moreover, Cibicek et al. [102] demonstrated that in acute dextran sulfate sodium (DSS)-induced rat colitis, isoquercitin dose-dependently ameliorated whole colon shortening and mitigated the DSS-induced expression of cyclooxygenase-2 and inducible nitric oxide synthase in the descending segment of the organ. However, when different parts of colon were assessed histomorphometrically, the results did not globally support the protective role of this flavonoid. In fact, the histological score was based on eight selected parameters that, when summarized, confirmed in DSS-treated rats a significant damage to colorectal mucosal tissue escalating in the aboral direction. However, the calculated index revealed no statistically significant effect of isoquercitrin on DSS induced colitis at either of the doses applied. The two most affected gut segments, i.e., the descending colon and rectum, were subjected to a more detailed statistical treatment. Focusing on edema, the trend observed in the descending colon reflected the gravimetrical data. In the rectum, however, the protective tendencies observable in the case of crypt dilatation, abscesses, level of inflammatory infiltrate and vascular congestion were counteracted by the remaining parameters. Thus, the rectum failed to display any consistent trend towards isoquercitrin generated protection, meaning that protective effects of flavonoids on acute enteritis depends on the site and the severity of tissue damage. Indeed, tissue healing trends observable in the descending colon were not apparent in the rectum, where histological damage was most severe. In a study the in vitro suppressive properties of selected polyphenols for dendritic cells’ production of inflammatory cytokines were tested and compared [88]. The in vitro suppressive properties of selected polyphenols for dendritic cells’ production of inflammatory cytokines have been tested and compared [103]. Then, a combination of quercetin and piperine were encapsulated into reconstituted oil bodies (ROBs) in order to increase their stability and administered to mice affected by a DSS-induced colitis. The results showed that administration of low dose polyphenol ROBs inhibited LPS-mediated inflammatory cytokine secretion, including IL-6, IL-23, and IL-12, while increasing IL-10 and IL-1Rα production. Mice administered with the polyphenol-containing ROBs were partially protected from DSS-induced colitis and associated weight loss, while mortality and inflammatory scores revealed an overall anti-inflammatory effect that was likely mediated by impaired dendritic cells’ immune responses. As regard resveratrol, its protective effect on the intestinal barrier has been previously tested in piglets with rotavirus-induced acute diarrhea [104]. Following pre-treatment with resveratrol dry suspension added to the basal diet for three weeks, piglets were orally challenged with rotavirus (RV). Researchers found that resveratrol alleviated RV-induced diarrhea, by inhibiting TNF-α production, thus reducing the inflammatory response, and maintaining the immune function, expressed as CD4+/CD8+ ratio. A recent trial also investigated whether dietary supplementation with resveratrol and curcumin could change the intestinal microbiota and alleviate intestinal inflammation induced by weaning in piglets [105]. Piglets were fed a control diet or a supplemented diet with a combination of antibiotics (olaquindox, kitasamycin, and chlortetracycline), or different dosage of either resveratrol and curcumin alone, or in combination for 28 days. The results showed that curcumin alone and antibiotics decreased the copy numbers of *E. coli* compared to controls. Both curcumin and resveratrol down-regulated the level of Toll-like-receptor 4 mRNA and protein expression in the intestine that results in the inhibition of the critical inflammation molecules release, and in the increase of immunoglobulin secretion. These results suggested that curcumin and resveratrol could regulate weaned piglet gut microbiota, alleviate intestinal inflammation, and ultimately increase intestinal immune function. Clinicopathological effects of phenolic compounds administration to animal models of AD are summarized in Table 3.

**Figure 2:**
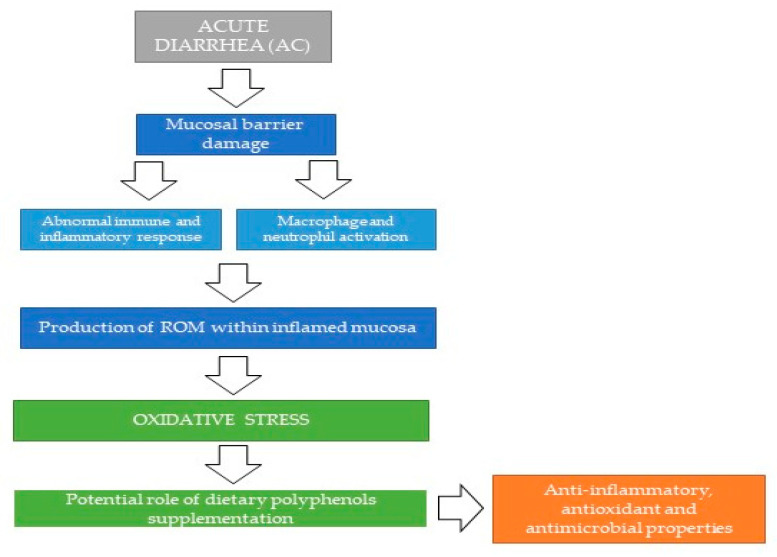
Possible mechanisms proposed for the beneficial role of polyphenols supplementation in the course of acute enteropathies. ROM: Reactive Oxygen Metabolites.

In particular, polyphenols can be useful in modulating the gut microbial ecosystem, limiting the translocation of pathogenic bacteria and reducing the inflammation associated with colonic mucosal damage. Possible mechanisms proposed for the beneficial role of polyphenols supplementation in the course of intestinal diseases are depicted in Figure 2.

Despite the results of the above-mentioned studies, clinical trials on human patients and on pet animals are scattered, and mainly focused on the effects on health conditions generally associated with oxidative stress and inflammation, rather than acute diarrhea [106].

## 4. Concluding Remarks

At present, a standardized protocol treatment for canine AD is lacking and most of the literature deals with clinical trials based on a combination of both therapeutic and nutritional interventions, for which the respective positive role is undeterminable. Studies evaluating the effects of different dietary approaches in course of acute enteropathies are limited exclusively to parvovirus enteritis [42]. Nevertheless, a nutritional approach is pivotal to canine AD management.

Antibacterials still represent a treatment option for AD, although both in human and veterinary medicine it has been widely demonstrated that their administration in uncomplicated cases is often unnecessary and negatively impacts the gut microbiota composition [6]. Thus, it is generally suggested that their use should be limited only in those cases presenting sepsis or at clear risk of sepsis [6,10,54,55].

Taking into account the well documented role played by oxidative stress in the pathogenesis of intestinal disorders, including acute diarrhea, studies in the canine patients should be encouraged in order to explore whether polyphenol supplementation may represent an effective anti-inflammatory and anti-oxidative integrative strategy to manage acute intestinal inflammation. Considering the peculiar mechanism of action and biotransformation process that distinguish phenolic molecules from other antioxidants, the former may act as postbiotic molecules, mainly in the colon, interfering with distribution and differentiation of microbial population. However, when considering appropriate treatment protocols, species differences in xenobiotic metabolism and microbiota should not be underestimated [66,73,74,77,78,79,104,105,106]. Given these limitations, properly targeted clinical trials dealing with the role of oxidative stress and polyphenol supplementation in both small and large bowel enteropathies in dogs are needed—especially in case of haemorrhagic diarrhea.

Finally, the availability of alternative treatment options, and hopefully of globally accepted guidelines, could assist practitioners in managing canine AD, while reducing inappropriate interventions and the risk of antibacterial resistance as well as significantly improving clinical outcomes.

## Figures and Tables

**Table 1:** Key nutritional factors for dogs with acute gastroenteritis. Modified from [35].

Factors	Recommended Levels *
Energy density	4.0–4.5 kcal/g
Fat	12–25% (highly digestible food); 8–12% (increased fiber-food)
Fiber	≤5% in highly digestible food (mixed fiber source preferred); 7–15% in fiber-enhanced food (insoluble or mixed fiber source preferred)
Digestibility	≥87% for protein and ≥90% for fat and carbohydrate (highly digestible food) ≥80% for protein and ≥90% for fat and carbohydrate (fiber-enhanced food)
Sodium	0.3–0.5%
Chloride	0.5–1.3%
Potassium	0.8–1.1%

* Nutrient levels are on a dry matter (DM) basis.

**Table 2:** Effects of polyphenols on gut microbiota. ↑ indicates an increase in bacteria population; ↓ indicates a decrease in bacteria population.

Phenolic Compound	Model	Effect on Gut Microbiota	Reference
tea phenolics (epicatechin, catechin, 3-*O*-methylgallic acid, gallic acid and caffeic acid)	Culture broth and culture broth with additional 0.1% (*w/v*) phenolic compounds inoculated with a 5% (*v/v*) bacterial inoculum and incubated at 37 °C under aerobic and anaerobic conditions	↓*Clostridium perfringens*, *Clostridium difficile* and *Bacteroides spp.* No effect on *Clostridium spp., Bifidobacterium spp*. and *Lactobacillus spp.*	[95,97]
proanthocyanidin- rich red wine extracts	rats	↑*Bacteroides*, *Lactobacillus* and *Bifidobacterium* spp. *Bacteroides*, ↓*Clostridium* and *Propionibacterium* spp.	[96,97]
(−)epicatechin and (+)catechin	batch-culture model, reflective of the distal region of the human large intestine	↑*E. coli*, *Clostridium coccoides–Eubacterium rectale* group ↓*Clostridium histolyticum* No effect *on Bifidobacterium* and *Lactobacillus* spp.	[98]
Wine polyphenols (4.4% anthocyanins, 0.8% flavonols, 2.0% phenolic acids, 1.4% catechin, 1.0% epicatechin and 28.0% proanthocyanidin units, consisting of 18.0% epigallocatechin, 13.2% catechin, 65.0% epicatechin and 3.8% epicatechin gallate)	F344 rats	↑*Bacteroides*, *Lactobacillus* and *Bifidobacterium* spp.	[99]

**Table 3:** Clinicopathological and histological effects of polyphenols in different in vivo models. ↑ indicates an increase; ↓ indicates a decrease.

Phenolic Compound	In Vivo Model	Effect	Reference
Quercitrin	Rat with trinitrobenzene sulfonic acid-induced colitis.	↓myeloperoxidase and alkaline phosphatase levels, counteract glutathione depletion, preserve normal fluid absorption and ↓ colonic damage.	[100]
Quercitin	Rat with trinitrobenzene sulfonic acid-induced colitis.	downregulate the inflammatory cascade associated with ↓of the disturbances in hydro-electrolytic transport.	[101]
Isoquercitin	Rat with acute dextran sulfate sodium (DSS)-induced colitis.	Dose-dependent ↓ of colon shortening and mitigation of DSS-induced expression of cyclooxygenase-2 and inducible nitric oxide synthase in the descending colon; protective effects depend on the site and the severity of tissue damage.	[102]
Quercetin + piperine, encapsulated into reconstituted oil bodies (ROBs)	Mice with acute dextran sulfate sodium (DSS)-induced colitis.	↓ LPS-mediated inflammatory cytokine secretion (IL-6, IL-23, and IL-12); ↑ IL-10 and IL-1Rα production. ↓ weight loss, mortality and inflammatory scores.	[104]
Resveratrol alone or with curcumin	Piglets orally challenged with rotavirus (RV).	alleviate RV-induced diarrhea, by ↓TNF-α production; down-regulate the level of Toll-like-receptor 4 mRNA and protein expression in the intestine; ↓ the critical inflammation molecules release, and immunoglobulin secretion.	[105]
